# Positron Emission Tomography (PET) with ^18^F-FGA for Diagnosis of Myocardial Infarction in a Coronary Artery Ligation Model

**DOI:** 10.1155/2022/9147379

**Published:** 2022-02-09

**Authors:** Vibhudutta Awasthi, Hariprasad Gali, Andria F. Hedrick, Huining Da, Venkateswararao Eeda, Diwakar Jain

**Affiliations:** ^1^College of Pharmacy, University of Oklahoma Health Sciences Center, Oklahoma City, Oklahoma, USA; ^2^Hexakit, Inc., 505 NE 46th, Oklahoma City, Oklahoma, USA; ^3^Westchester Medical Center, 100 Woods Road, Valhalla, NY, USA

## Abstract

Location and extent of necrosis are valuable information in the management of myocardial infarction (MI). *Methods*. We investigated 2-deoxy-2-^18^F-fluoro glucaric acid (FGA), a novel infarct-avid agent, for positron emission tomography (PET) of MI. We synthesized FGA from commercially available ^18^F-fluoro-2-deoxy-2-D-glucose (FDG). MI was induced in mice by permanently occluding the left anterior descending coronary artery. Biodistribution of FGA was assessed 1 h after FGA injection (11 MBq). PET/CT was conducted 1 h, 6 h, 1 d, 3 d, and 4 d after MI. Subcellular compartment of FGA accumulation in necrosis was studied by tracing the uptake of biotin-labeled glucaric acid with streptavidin-HRP in H_2_O_2_-treated H9c2 cardiomyoblasts. Streptavidin-reactive protein bands were identified by LC-MS/MS. *Results*. We obtained a quantitative yield of FGA from FDG within 7 min (radiochemical purity > 99%). Cardiac uptake of FGA was significantly higher in MI mice than that in control mice. Imaging after 1 h of FGA injection delineated MI for 3 days after MI induction, with negligible background signal from surrounding tissues. Myocardial injury was verified by tetrazolium staining and plasma troponin (47.63 pg/mL control versus 311.77 pg/mL MI). In necrotic H9c2 myoblasts, biotinylated glucaric acid accumulated in nuclear fraction. LC-MS/MS primarily identified fibronectin in necrotic cells as a putative high fidelity target of glucaric acid. *Conclusion*. FGA/PET detects infarct early after onset of MI and FGA accumulation in infarct persists for 3 days. Its retention in necrotic cells appears to be a result of interaction with fibronectin that is known to accumulate in injured cardiac tissue.

## 1. Introduction

Myocardial infarction (MI) is a leading cause of death in developed nations [1]. The clinical goal in these patients is to salvage viable tissue by restoring blood supply to ischemic regions and prevent infarction in at-risk tissue. Thus, MI requires rapid and accurate diagnosis for effective thrombolytic therapy or revascularization [2]. The initial diagnosis of MI depends on an electrocardiogram and circulating biomarkers. Among circulating biomarkers, cardiac troponin (cTnI) is a sensitive indicator of MI. It increases within 4-6 h, peaks at 24 h, and can remain elevated for 1-2 weeks [3]. To confirm MI diagnosis, input from single-photon emission computed tomography (SPECT) or positron emission tomography (PET) is routinely employed with radiotracers such as ^99m^Tc-Sestamibi, ^201^Tl, ^99m^Tc-tetrofosmin, ^13^N-ammonia, ^82^Rb-RbCl_2_, and ^18^F-Flurpiridaz [4]. These radiotracers are classified as perfusion agents because their tissue accumulation is perfusion-dependent, implying only viable tissue shows up in images and dead tissue remains dark. Both PET and SPECT perfusion imaging techniques can locate and quantify perfusion deficit in the myocardial space of epicardial coronary artery diseases with high accuracy. However, detection of necrotic/dead tissue and the extent of infarction is largely based on the principle of elimination, and perfusion scans cannot discriminate between ischemic and necrotic regions because both show reduced perfusion. Infarct-avid agents are an emerging class of imaging agents that accumulate in the necrotic area, thus providing direct detection of infarct tissue and clear distinction between ischemic and necrotic regions of the heart. Since tissue necrosis starts within the first few hours of coronary occlusion, information obtained from direct imaging of necrotic tissue in conjunction with perfusion imaging could be of immense value in the evaluation of MI patients [5].

Because of higher count rate and less tissue attenuation, PET is a more sensitive nuclear medicine modality as compared to SPECT, which makes PET an attractive modality for MI diagnosis [6, 7]. Whereas PET radiotracers for metabolism [8] and perfusion [9, 10] exist, a radiotracer for direct imaging of myocardial infarction is not available. We recently reported ^18^F-labeled glucaric acid (FGA) as the first infarct-avid PET agent [11, 12]. FGA was designed based on previous observations that ^99m^Tc-labeled glucarate avidly accumulates in infarcted myocardium for SPECT imaging of MI [5, 13]. The objectives of this study were to evaluate the biodistribution and PET imaging of ^18^F-FGA in a mouse model of MI and to identify its intracellular binding partners in necrotic cells.

## 2. Materials and Methods

All reagents were purchased from Sigma-Aldrich or VWR International (Radnor, PA). ^18^F-Fluorodeoxyglucose (FDG) was from SOFIE (Dallas, TX). CD1 mice were obtained from Charles River Laboratories (Wilmington, MA). Animal studies were compliant with the ARRIVE and AVMA guidelines and approved by the Institutional Animal Care and Use Committee of the University of Oklahoma Health Sciences Center.

### 2.1. Synthesis of ^18^F–FGA

^18^F-FGA was synthesized from ^18^F-FDG using 3-vial kits provided by Hexakit, Inc. Briefly, 2 mL of ^18^F-FDG (574-1040 MBq) was added to vial A, followed by addition of 60 *μ*L of NaOCl solution (supplied as vial B). After 7 min, the reaction was stopped by adding 100 *μ*L of buffer (supplied as vial C). The reaction mixture was filtered through a 0.2 *μ*m filter and subjected to radio-thin layer chromatography (TLC) to ensure that ^18^F-FDG is quantitatively converted into ^18^F-FGA. Briefly, the sample was run on a plastic-backed silica gel using a 9 : 1 *v*/*v* acetonitrile : water system. The TLC strip was read on a miniscan 1000 (Bioscan, Washington DC). The product was further evaluated by High-Performance Liquid Chromatography (HPLC) as described earlier [11].

### 2.2. MI Model

We used a mouse model that creates an infarct mimicking the coronary artery blockage produced by coronary artery disease in humans [14]. CD1 mice (25-27 g, female, 6-8 weeks) were acclimatized for at least 5 days under a standardized light/dark cycle and allowed *ad libitum* access to rodent chow and tap water. Mice were anesthetized and endotracheally intubated for ventilation with 1.5% isoflurane-O_2_ stream. A 1-5 cm incision was made in the 4^th^-5^th^ intercostal space. The pericardium was opened to locate and permanently occlude the left anterior descending (LAD) artery using 8-0 nylon suture. After closing the incision, the mice were kept on O_2_-ventilator until spontaneous breathing resumed. Saline (0.2 mL i.p.) and buprenorphine (1 mg/kg s.c.) were administered for hydration and analgesia, respectively.

### 2.3. PET/CT Imaging

PET/CT were conducted using a Gamma Medica-Ideas' PET/CT machine (Northridge, CA). Mice were injected with ^18^F-FGA (5.5-18.5 MBq/0.3 mL i.v.) and imaged after 1 h. PET data was acquired over a period of 10 min followed by CT imaging for 2 min. The data were reconstructed by filtered back projection, and the images were fused with CT images. Standard uptake value (SUV) was calculated by defining a region of interest (ROI) using Amira 5 software (ThermoFisher, Waltham, MA). Background ROI was drawn at the 4^th^ sternebrae in the *XY* plane of the posterior medial area of the left lung.

### 2.4. Biodistribution

A biodistribution study was carried out according to the methods described earlier [11]. Mice with or without MI were anesthetized with 2% isoflurane-O_2_ and injected with a dose of ~0.37 MBq of 18F-FGA in 50-100 *μ*L through a tail vein. The mice were euthanized at 15 min, 1 h, or 2 h postinjection (p.i.) by isoflurane overdose and cervical dislocation to collect blood, urine, and various tissues/organs. The radioactivity associated with tissues was corrected for radioactive decay, and uptake was expressed as injected dose per gram (%ID/g). Total blood volume, bone, and muscle mass were estimated as 5.7%, 10%, and 40% of body weight, respectively.

### 2.5. Histology

The heart was sliced into 1-2 mm sections, and the sections were stained with 1% solution 2,3,5-triphenyltetrazolium chloride (TTC). After 20 min of staining, the sections were fixed in 10% buffered formalin.

### 2.6. Cardiac Troponin I (cTnI) Assay

We determined cTnI in 1 : 2 diluted plasma samples by an enzyme-linked immunoassay kit (Life Diagnostics, West Chester, PA). The manufacturer-suggested protocol was followed.

### 2.7. H9c2 Cell Culture and FGA Uptake

H9c2 rat myoblasts (ATCC, Manassas, VA) were cultured in Dulbecco's modified Eagle's medium with FBS (10%), penicillin (100 IU), streptomycin (100 *μ*g/mL), and sodium pyruvate (1 mM) at 37°C in 5% CO_2_ environment. It is an immortalized cardiomyoblast line derived from an embryonic rat heart. Although myoblastic, it maintains the potential to mature into myocytes in culture. We validated the differentiation conditions for H9c2 cells (Supplemental Figure S1). Confluent H9c2 cells were treated with 50 *μ*M H_2_O_2_ and 10 mM biotinylated-glucaric acid (BGA) for 30 min. Control cells were treated with BGA only. BGA synthesis is described in the supplement (Scheme S1). Cells were harvested, homogenized, and fractionated into nuclear, mitochondrial, and cytosolic fractions using a kit (abcam, Cambridge, MA). These fractions were electrophoretically separated on 4-20% polyacrylamide gels and transferred to nitrocellulose membranes for blotting with streptavidin-HRP (BD Pharmingen, San Jose, CA). The bands were detected by ECL Substrate (Bio-Rad, Hercules, CA). To ensure the fraction purity, the membranes were immunoprobed for GAPDH (cytosolic marker), COX IV (mitochondrial marker), and histone (nuclear marker).

### 2.8. Pull-Down Assay and Target Identification

Nuclear fraction of H_2_O_2_-treated and BGA-labeled H9c2 cells was used for protein pull-down assay using monomeric-avidin (G-Biosciences, St. Louis, MO). In brief, 700 *μ*g protein was incubated with avidin beads for 1 h with constant rotation. The beads were pelleted by centrifugation, washed, and avidin-bound proteins were eluted by 2 mM biotin. The samples were subjected to streptavidin-HRP blotting as described above. To identify the binding partner of BGA, the proteins pulled down by avidin beads were submitted to Kendrick Laboratories (Madison, WI) for LC-MS/MS. The LC-MS/MS processing methods are described elsewhere [15–19] and reported in supplement. The protein database search was performed against NCBI all organisms.

### 2.9. Data Analysis

Comparisons were made by 2-tailed Student's *t*-test for two-sample unequal variance (*p* < 0.05). Radioactivity counts were corrected for decay half-life (110 min). Densitometry of blots was performed by NIH's Image J software. SUVs were calculated by multiplying the heart ROI activity concentration (MBq/mL) by mouse weight (g) and then dividing by injected dose (MBq).

## 3. Results

**Figure 1:**
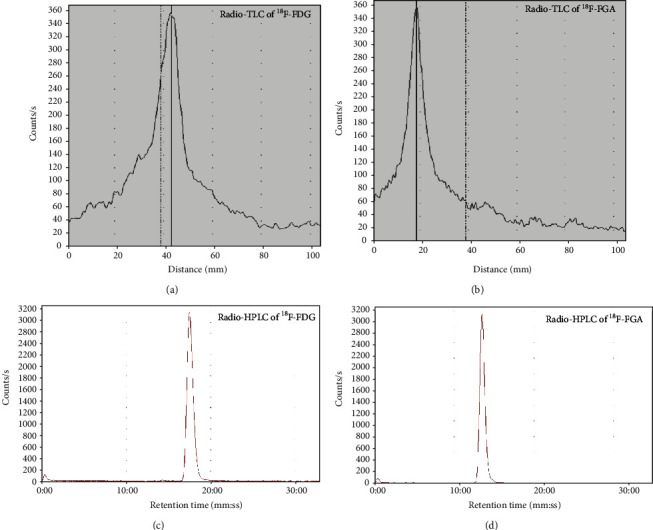
Quality control of ^18^F-FGA. (a) and (b) are radio-TLC profiles of ^18^F-FDG and ^18^F-FGA, respectively. (c) and (d) are radio-HPLC chromatograms of ^18^F-FDG and ^18^F-FGA, respectively.

^18^F-FGA was synthesized by controlled oxidation of ^18^F-FDG. Radiochemical purity > 99% of ^18^F-FGA was obtained on a consistent basis and within 5-7 min of oxidation reaction. In TLC, ^18^F-FDG showed *R*_*f*_ = 0.64, and ^18^F-FGA showed *R*_*f*_ = 0.16. In HPLC, ^18^F-FDG and ^18^F-FGA analytes showed well-separated retention times of approximately 13.4 min and 17.5 min, respectively (Figure 1).

### 3.1. PET/CT Imaging

**Figure 2:**
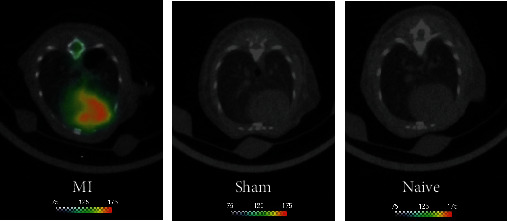
^18^F-FGA/PET of MI in mice. Mice belonging to MI, sham, and naïve groups were injected with 300 *μ*Ci of ^18^F-FGA. PET was performed after 1 h. Only MI mice showed uptake in the heart; sham and naïve mice did not show any detectable uptake in the heart.

Figure 2 shows a representative set of PET/CT images after 3 h of MI induction; images were acquired after 1 h of ^18^F-FGA injection. Mice with MI showed cardiac uptake of ^18^F-FGA, but sham and naïve mice showed no detectable accumulation in the field of view. In addition to its selectivity for infarcted heart compared to normal healthy heart, ^18^F-FGA uptake in the liver was negligible. The myocardial uptake of ^18^F-FGA determined at necropsy after 1 h of injection was 0.42% ID/g in MI versus 0.06% ID/g in normal mice (*p* < 0.05). The clearance of ^18^F-FGA from blood was rapid; almost all excreted radioactivity was detected in the urine.

**Figure 3:**
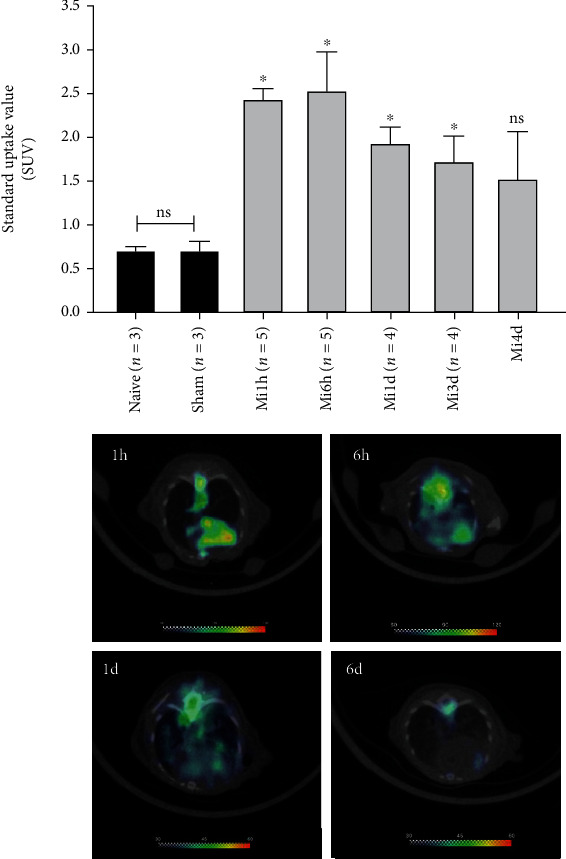
Cardiac SUV of ^18^F-FGA at various times after MI (mean ± sem; ^∗^*p* < 0.05 vs. sham). The lower panel shows representative tomograms of mice imaged after 1 h, 6 h, 1 d, and 3 d after MI.

**Figure 4:**
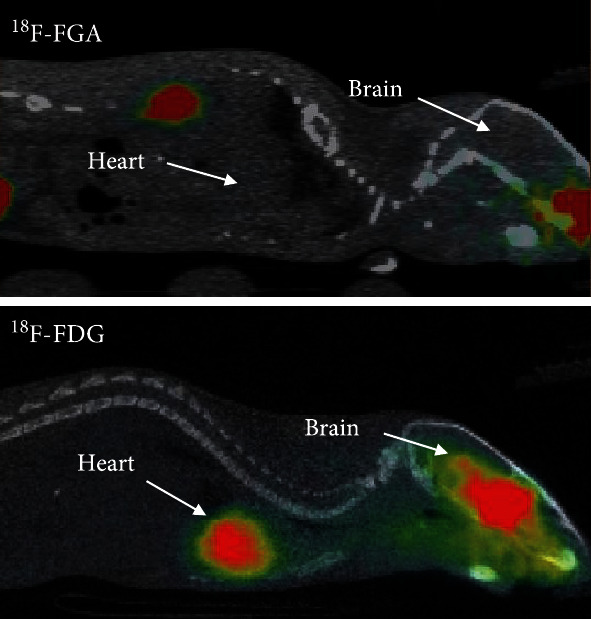
Whole-body images of mice injected with ^18^F-FGA and ^18^F-FDG. Approximately 10 MBq of ^18^F-FGA and ^18^F-FDG was intravenously injected in normal mice, and PET images were acquired after 30 min of injection. Whereas ^18^F-FGA rapidly cleared from circulation via the kidneys and did not show any accumulation in the normal heart and brain, ^18^F-FDG was significantly retained in the heart and brain.

Next, we determined the optimal imaging time post-MI by imaging on longitudinal basis. The mice were subjected to ^18^F-FGA/PET imaging at various times after MI induction, i.e., 1 h (*n* = 5), 6 h (*n* = 5), 1 d (*n* = 4), 3 d (*n* = 4), and 4 d (*n* = 4) post-MI. Naïve and sham groups were represented by three mice each. As shown in Figure 3, uptake of ^18^F-FGA remained significantly higher in MI heart than in control heart for up to 3 days. However, the absolute uptake of ^18^F-FGA in MI heart declined with time post-MI. These data indicated that ^18^F-FGA/PET could remain positive for 3 days after MI. It is noteworthy that compared to ^18^F-FDG, ^18^F-FGA does not accumulate in the normal heart and brain (Figure 4).

### 3.2. Validation of the MI Model

**Figure 5:**
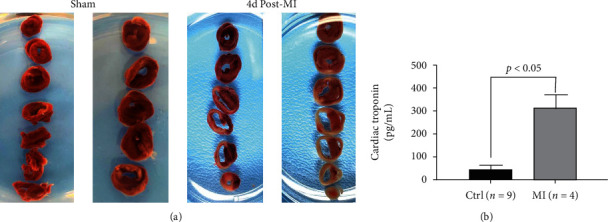
Validation of MI model. (a) TTC-stained slices of sham and MI hearts. MI hearts showed significant thinning of the ventricular wall and large areas of unstained necrotic tissue. (b) Cardiac troponin levels in plasma from sham control versus MI mice.

As shown in Figure 5(a), LAD ligation created a significant histologic injury. TTC stained the healthy tissue in red color, whereas infarcted tissue remained as unstained or pale. We also noted considerable thinning of the ventricular wall in the MI group. The mice with MI also showed significantly higher levels of cTnI in plasma as compared to the control group (Figure 5(b)). These data validated our model employed for imaging of MI.

### 3.3. Tissue Biodistribution

Biodistribution of ^18^F-FGA in normal and MI mice after 1 h of ^18^F-FGA injection is given in Table 1. The blood clearance of ^18^F-FGA was rapid, and almost all ^18^F-FGA was excreted in urine by 2 h p.i. More importantly, less than 0.6%ID/g of ^18^F-FGA was found to be collectively associated with the blood, heart, and liver at 1 h p.i. These data suggested that ^18^F-FGA does not accumulate in a normal healthy heart, and its negligible liver uptake would not obfuscate MI imaging. In contrast to the lack of uptake in the healthy heart of normal mice, ^18^F-FGA showed significantly high uptake in the MI heart; the uptake was 7.5 times more in the MI heart than in the normal heart. Additional biodistribution data of ^18^F-FGA in normal healthy mice at 15 min and 2 h postinjection is provided in Supplemental Table S2.

### 3.4. Glucaric Acid Accumulation in Nuclear Fraction of Oxidatively Stressed H9c2 Cells

**Figure 6:**
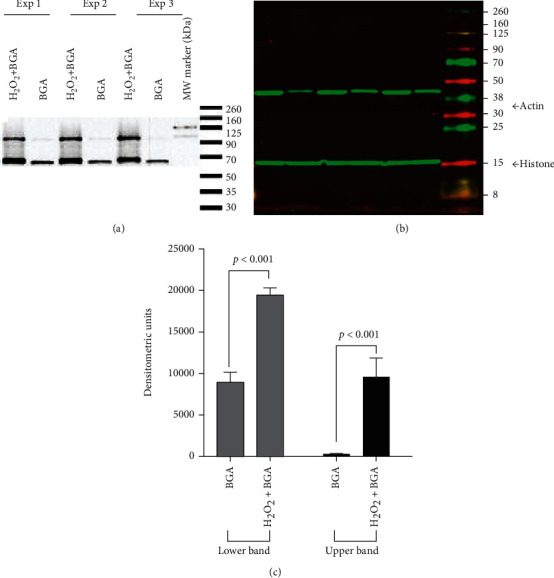
Nuclear localization of biotinylated glucaric acid (BGA) in necrotic H9c2 cells. (a) Streptavidin-HRP blot of nuclear fraction. (b) Immunoblots of histone (15 kDa) and actin (43 kDa). (c) Densitometry (DM; *n* = 3) of two streptavidin-reactive bands.

To mimic necrotic cell death in MI, we employed an *in vitro* model of H_2_O_2_-induced necrosis in H9c2 cardiomyoblasts. H9c2 cells differentiate into myocytes under Pagano et al.'s culture conditions [20] as described in Supplemental Figure S1c. The onset of death in H_2_O_2_-treated H9c2 cells was characterized by altered cellular morphology. As previously reported [11], H_2_O_2_-treated H9c2 cells accumulated significantly higher amounts of ^18^F-FGA as compared to normal H9c2 cells. Here, we used a biotinylated analog of glucaric acid to track its accumulation in necrotic cells. We found enhanced localization of BGA in the nuclear fraction of H_2_O_2_-treated cells as compared to that of control cells (Figure 6(a)). Western blotting of H9c2 nuclear fraction showed two immunoreactive bands of approximate molecular mass 120 kDa and 80 kDa. The densities of upper and lower bands were 30 and 2.2 times in H_2_O_2_-treated cells than in control H9c2 cells, respectively (Figure 6(c)). Cytoplasmic fractions of H_2_O_2_-treated and control H9c2 cells showed no difference (Supplement Figure S3). Interestingly, we also observed that in the mitochondrial fractions, the protein bands of the same molecular weight reduced in intensity after H_2_O_2_ treatment (Supplemental Figure S3).

**Figure 7:**
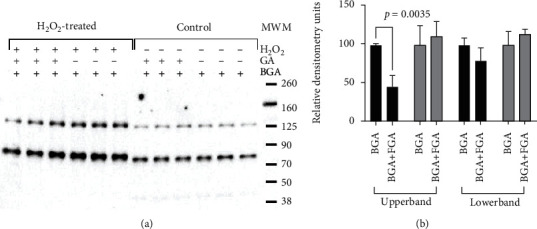
Competition of glucaric acid (GA) with biotinylated glucaric acid (BGA) for binding with nuclear proteins. (a) A representative streptavidin-HRP blot. (b) Densitometry of streptavidin-reactive bands (*n* = 3).

When nuclear fractions from H_2_O_2_-treated H9c2 cells were treated with excess of free unlabeled glucaric acid before adding BGA, the band intensity of BGA-interacting target protein was reduced (Figure 7(a)). Only protein of higher molecular mass (120 kDa) was significantly affected, whereas difference in lower molecular mass protein was not significant (Figure 7(b)). This indicated the specific nature of interaction between BGA and target protein(s). Interestingly, BGA did not interact with nuclear fractions of normal cells and this was not affected by competing free glucaric acid.

Having realized that the target protein for interaction with BGA resides in the nuclear fraction, we eluted the protein pulled down by BGA-avidin beads and separated the samples on an electrophoretic gel. The characteristic 120 kDa and 80 kDa bands were excised for LC-MS/MS. The database search of mass spectroscopy results projected three protein identities of fibronectin, nonmuscle heavy chain myosin II-A, and filamin-A isoform X2 for 120 kDa band and actinin-4 (ACTN4) for 80 kDa band (Table 2). The identity of fibronectin was associated with a high Mascot score of 147 and peptide identity of 12, suggesting it to be the most likely intracellular partner for interaction with glucaric acid analogs.

## 4. Discussion

We evaluated ^18^F-FGA for detection of MI in a surgical model and investigated the subcellular fraction responsible for the accumulation of glucaric acid in necrotic cells. The major findings of this study were as follows: (i) ^18^F-FGA selectively accumulates in infarcted myocardium while rapidly clearing from the rest of the body, (ii) rapid washout of ^18^F-FGA meant that the optimal imaging time after ^18^F-FGA injection is 1 h p.i., (iii) ^18^F-FGA localization in injured myocardium is highest early after occlusion, but significant uptake persists even after 3 days of MI, and (iv) fibronectin is the likely target of glucarate analogs in necrotic myocardial cells.

We previously reported that ^18^F-FGA/PET sensitively detected brain stroke [12] and isoproterenol-induced cardiomyopathy [11], demonstrating ^18^F-FGA as the first potential PET radiotracer for the detection of infarct in acute clinical setting. Among SPECT radiotracers, ^111^In-antimyosin, ^99m^Tc-pyrophosphate, and ^99m^Tc-glucarate could be recognized as competing radiotracers for ^18^F-FGA. However, none of these is available for routine clinical work up. ^111^In-antimyosin and ^99m^Tc-pyrophosphate suffer from a drawback of extracardiac accumulation in the liver and bone, respectively. ^111^In-antimyosin also persists in circulation for several days, which makes it difficult to use in acute setting. Uptake in surrounding tissues or high background signal from blood can cause artifacts in cardiac imaging, making diagnostic interpretation difficult [21, 22]. In contrast, there is no accumulation of ^18^F-FGA in the liver, lung, or bone (Supplemental Table S2). Being a highly water-soluble small molecule (MW = 227), ^18^F-FGA is rapidly cleared by the renal system. Furthermore, as opposed to ^111^In-antimyosin and ^99m^Tc-pyrophosphate, the uptake of ^18^F-FGA in infarct occurs rapidly after onset of MI. SPECT analog ^99m^Tc-glucarate also accumulates in MI within 30 min post-MI [23], but it delineates MI in patients only if imaging is performed within 9 h of chest pain [24]. Our findings indicate that ^18^F-FGA not only diagnoses MI early but also through 3 days post-MI. This longitudinal success makes ^18^F-FGA/PET different from ^99m^Tc-glucarate/SPECT.

Despite the knowledge about ^99m^Tc-glucarate accumulation in MI since 1990s, the molecular basis of glucarate uptake in infarct remains unknown and there is no consensus on the mechanism of its transport and molecular target in necrotic cells. Initially, it was speculated that glucarate permeates through the leaky cell membrane of necrotic cells and electrostatically binds to the positively charged nuclear histone proteins [23, 25, 26]. However, no evidence was provided to support glucarate-histone interaction. An alternative hypothesis of the involvement of fructose transporter GLUT5 in the uptake of ^99m^Tc-glucaric acid by cancer cells was proposed [27–29], which has been recently supported by an experimental observation that excess fructose reduces the uptake of ^18^F-FGA in lung, breast, and brain cancer cell lines [30, 31]. However, GLUT5-mediated transport does not explain the accumulation of ^18^F-FGA in acute MI because GLUT5 is not expressed in the cardiac tissue [32, 33]. Our data suggests that glucaric acid localizes in nuclei of necrotic cells and most likely interacts with fibronectin. Whether the source of fibronectin in MI tissue is plasma-derived, platelet-derived, or de novo synthesis in response to injury is subject to further investigation. Similarly, the rationale behind the association of fibronectin with nuclear fraction of necrotic cells remains unclear. Literature is also silent on this aspect, but a study by Savill and Ayad suggested that fibronectin or fibronectin degradation products could be taken up by the nuclei of cultured cells [34].

Fibronectin is a glycoprotein and a component of extracellular matrix. It is particularly important for heart development during embryogenesis as cardiomyocytes, fibroblasts, and endothelial cells migrate on fibronectin. After embryogenesis, fibronectin amount in cardiac muscle decreases, and only negligible presence is seen in healthy adult hearts [35]. In a failing heart, the distribution and amount of the extracellular matrix proteins and cytoskeletal elements change [36]. Several preclinical and clinical studies have given immunohistochemical evidence that expression of fibronectin in the heart increases secondary to the tissue-repair processes induced after MI. The expression of fibronectin receptor, integrin *α*5*β*1 also increases during the healing process both in peri-infarcted and noninfarcted areas [37]. In a rat model of coronary artery ligation, Casscells et al. found increased intracellular immunochemical staining for fibronectin in the necrotic myocytes within 4 h after MI, peaking in intensity at 48 h and declining after 72 h; viable myocytes did not show any fibronectin immunoreactivity at all stages of infarction [38]. A rapid and progressive increase in cardiac fibronectin mRNA and protein expression in the infarcted region of the ventricle was also reported in a rabbit model of MI [39]. Holmbom et al. found close correspondence of antifibronectin staining with the infarct size from tetrazolium-stained slices in a pig MI model [40]. More recently, fibronectin expression was identified as one of the early markers for identifying MI as a cause of death in human post mortem samples [41]. In MI patients, fibronectin appears in the ischemic cardiomyocytes within a day and disappears gradually as the scar tissue develops [42]. A specific increase in cardiac fibronectin expression has also been reported in patients with myocarditis [43–45]. Furthermore, deposition of fibronectin was also observed in cardiomyocytes of posttransplant heart injury, and fibronectin positivity in cardiac tissue was reported to correlate very closely with the appearance of necrosis [46]. These reports clearly indicate that fibronectin accumulates in MI, but its accumulation has never been exploited for diagnosis or staging of patients. We found selective interaction of glucaric acid with a 120 kDa protein of necrotic cells. The molecular weight of immunoreactive protein matches with a reported fibronectin fragment of 120 kDa in the MI heart [47, 48]. Injury to cardiomyocytes causes activation of proteases that cleave fibronectin, releasing a 120 kDa fibronectin fragment within 3-5 h after MI [47, 48]. We suspect the other ~80 kDa protein is the remnant of the fibronectin molecule (fibronectin monomer is 204 kDa). Based on these results and published literature on fibronectin accumulation in MI, we hypothesize that the mechanistic target of FGA is fibronectin in cardiomyocytes undergoing necrosis.

## 5. Conclusions

^18^F-FGA/PET is a promising imaging technique that can combine with traditional perfusion imaging to increase diagnostic specificity in MI pathology. ^18^F-FGA is quickly synthesized, within minutes and in a quantitative manner from commercially and ubiquitously available ^18^F-FDG from nuclear pharmacies. A significant advantage of this method is that it does not require any work-up to isolate ^18^F-FGA. To our knowledge, except for ^18^F-FGA, there are no other PET agents for direct infarct imaging. Our pilot data are the first to demonstrate fibronectin interaction as a mechanistic basis of glucaric acid localization in MI and potential imaging of fibronectin accumulation in MI pathology. Knowing the intracellular proteins interacting with ^18^F-FGA will assist in the development of new agents with improved binding kinetics. However, the singular identity of interacting domain in fibronectin protein warrants further investigation.

## Figures and Tables

**Table 1:** Biodistribution of ^18^F-FGA in normal and MI mice after 1 h of injection (mean + SD; *n* = 6/group).

Organ	Normal		MI	
	%ID	%ID/g	%ID	%ID/g
Blood	0.25 ± 0.09	0.16 ± 0.05	0.72 ± 0.54	0.50 ± 0.40
Muscle	0.47 ± 0.17	0.04 ± 0.02	1.61 ± 0.80	0.15 ± 0.07
Bone	3.78 ± 3.50	1.37 ± 1.21	4.58 ± 3.24	1.81 ± 1.44
Brain	0.03 ± 0.04	0.06 ± 0.08	0.02 ± 0.01	0.05 ± 0.02
Heart	0.01 ± 0.00	0.06 ± 0.03	0.05 ± 0.02	0.42 ± 0.25
Lung	0.03 ± 0.01	0.15 ± 0.04	0.09 ± 0.04	0.53 ± 0.27
Liver	0.44 ± 0.12	0.31 ± 0.08	1.40 ± 0.84	0.31 ± 0.08
Spleen	0.01 ± 0.00	0.09 ± 0.04	0.01 ± 0.00	3.00 ± 3.66
Kidney	1.36 ± 0.26	3.70 ± 0.80	1.91 ± 0.98	5.49 ± 4.92
Stomach	0.10 ± 0.08	0.18 ± 0.20	0.28 ± 0.22	0.39 ± 0.26
Intestine	1.14 ± 0.42	0.47 ± 0.18	0.93 ± 0.26	0.37 ± 0.04

**Table 2:** Proteins interacting with glucaric acid as identified by nanoLC-MS/MS.

Protein identified	Mascot score	Peptide sequences identified
Fibronectin	147	12
Nonmuscle heavy chain myosin II-A	106	4
Filamin-A X2	80	4
*α*-Actinin-4	65	8

## Data Availability

The experimental data used to support the findings of this study are included within the article.

## Additional Points

*New and Noteworthy*. ^18^F-FGA is the first infarct-avid agent for PET imaging of myocardial infarction, which rapidly delineated myocardial injury in a mouse model. The likely target of ^18^F-FGA accumulation in necrotic cells is fibronectin. Implications for Patient Care: PET/^18^F-FGA could be a clinical diagnostic tool, particularly to detect the location and extent of infarct in the heart.

## Supplementary Materials

Supplementary Materials Supplemental information and original unedited pictures of Western blots are provided.

## References

[1] Mozaffarian D., Benjamin E. J., Go A. S. (2015). Heart disease and stroke statistics—2015 Update. *Circulation*.

[2] Allman K. C., Shaw L. J., Hachamovitch R., Udelson J. E. (2002). Myocardial viability testing and impact of revascularization on prognosis in patients with coronary artery disease and left ventricular dysfunction: a meta-analysis. *Journal of the American College of Cardiology*.

[3] McCann C. J., Glover B. M., Menown I. B. (2008). Novel biomarkers in early diagnosis of acute myocardial infarction compared with cardiac troponin T. *European Heart Journal*.

[4] Klein R., Celiker-Guler E., Rotstein B. H., deKemp R. A. (2020). PET and SPECT tracers for myocardial perfusion imaging. *Seminars in Nuclear Medicine*.

[5] Gerson M. C., McGoron A. J. (1997). Technetium 99m glucarate: what will be its clinical role?. *Journal of Nuclear Cardiology*.

[6] Parker M. W., Iskandar A., Limone B. (2012). Diagnostic accuracy of cardiac positron emission tomography versus single photon emission computed tomography for coronary artery disease: a bivariate meta-analysis. *Circulation. Cardiovascular Imaging*.

[7] Mc Ardle B. A., Dowsley T. F., deKemp R. A., Wells G. A., Beanlands R. S. (2012). Does Rubidium-82 PET Have Superior Accuracy to SPECT Perfusion Imaging for the Diagnosis of Obstructive Coronary Disease?: A Systematic Review and Meta- Analysis. *Journal of the American College of Cardiology*.

[8] Ghesani M., Depuey E. G., Rozanski A. (2005). Role of F-18 FDG positron emission tomography (PET) in the assessment of myocardial viability. *Echocardiography*.

[9] Di Carli M. F., Dorbala S., Meserve J., El Fakhri G., Sitek A., Moore S. C. (2007). Clinical myocardial perfusion PET/CT. *Journal of Nuclear Medicine*.

[10] Berman D. S., Maddahi J., Tamarappoo B. K. (2013). Phase II safety and clinical comparison with single-photon emission computed tomography myocardial perfusion imaging for detection of coronary artery disease: flurpiridaz F 18 positron emission tomography. *Journal of the American College of Cardiology*.

[11] Houson H. A., Nkepang G. N., Hedrick A. F., Awasthi V. (2018). Imaging of isoproterenol-induced myocardial injury with ^18^F labeled fluoroglucaric acid in a rat model. *Nuclear Medicine and Biology*.

[12] Houson H., Mdzinarishvili A., Gali H., Sidorov E., Awasthi V. (2020). PET detection of cerebral necrosis using an infarct-avid agent 2-Deoxy-2-[18F]Fluoro-d-Glucaric acid (FGA) in a mouse model of the brain stroke. *Molecular Imaging and Biology*.

[13] de Murphy C. A., Ferro-Flores G., Villanueva-Sanchez O. (2002). 99mTc-glucarate for detection of isoproterenol-induced myocardial infarction in rats. *International Journal of Pharmaceutics*.

[14] Xu Z. B., Alloush J., Beck E., Weisleder N. (2014). A murine model of myocardial ischemia-reperfusion injury through ligation of the left anterior descending artery. *JoVE*.

[15] Lux J. C., Channaveerappa D., Aslebagh R. (2019). Identification of dysregulation of atrial proteins in rats with chronic obstructive apnea using two-dimensional polyacrylamide gel electrophoresis and mass spectrometry. *Journal of Cellular and Molecular Medicine*.

[16] Mihasan M., Babii C., Aslebagh R., Channaveerappa D., Dupree E., Darie C. C. (2018). Proteomics based analysis of the nicotine catabolism in Paenarthrobacter nicotinovorans pAO1. *Scientific Reports*.

[17] Aslebagh R., Channaveerappa D., Arcaro K. F., Darie C. C. (2018). Comparative two-dimensional polyacrylamide gel electrophoresis (2D-PAGE) of human milk to identify dysregulated proteins in breast cancer. *Electrophoresis*.

[18] Wormwood K. L., Ngounou Wetie A. G., Gomez M. V. (2018). Structural characterization and disulfide assignment of spider peptide Ph*α*1*β* by mass spectrometry. *Journal of the American Society for Mass Spectrometry*.

[19] Channaveerappa D., Lux J. C., Wormwood K. L. (2017). Atrial electrophysiological and molecular remodelling induced by obstructive sleep apnoea. *Journal of Cellular and Molecular Medicine*.

[20] Pagano M., Naviglio S., Spina A. (2004). Differentiation of H9c2 cardiomyoblasts: the role of adenylate cyclase system. *Journal of Cellular Physiology*.

[21] Dvorak R. A., Brown R. K., Corbett J. R. (2011). Interpretation of SPECT/CT myocardial perfusion images: common artifacts and quality control techniques. *Radiographics*.

[22] Tamaki N., Yamada T., Matsumori A. (1990). Indium-111-antimyosin antibody imaging for detecting different stages of myocardial infarction: comparison with technetium-99m-pyrophosphate imaging. *Journal of Nuclear Medicine*.

[23] Narula J., Petrov A., Pak K. Y., Lister B. C., Khaw B. A. (1997). Very early noninvasive detection of acute experimental nonreperfused myocardial infarction with 99mTc-labeled glucarate. *Circulation*.

[24] Mariani G., Villa G., Rossettin P. F. (1999). Detection of acute myocardial infarction by 99mTc-labeled D-glucaric acid imaging in patients with acute chest pain. *Journal of Nuclear Medicine*.

[25] Narula J., Petrov A., Pak K. Y., Khaw B.-A. (1995). 962-122 very early noninvasive imaging of acute myocardial infarcts with Tc-99m-glucarate. *Journal of the American College of Cardiology*.

[26] Khaw B. A., Silva J. D., Petrov A., Hartner W. (2002). Indium 111 antimyosin and Tc-99m glucaric acid for noninvasive identification of oncotic and apoptotic myocardial necrosis. *Journal of Nuclear Cardiology*.

[27] Isnardi V., Clotagatide A., Bruel S., Perek N. (2012). Is [(99m)Tc] glucarate uptake mediated by fructose transporter GLUT-5?. *Nuclear Medicine and Biology*.

[28] Kannan S., Begoyan V. V., Fedie J. R. (2018). Metabolism-driven high-throughput cancer identification with GLUT5-specific molecular probes. *Biosensors*.

[29] Choudhury P. S., Alonso O., Dondi M. (2012). 99mTc glucarate as a potential radiopharmaceutical agent for assessment of tumor viability: from bench to the bed side. *World Journal of Nuclear Medicine*.

[30] Li J., Beverly L., Gray B., Pak K., Ng C. (2020). Assessment of 18F-fluoroglucaric acid uptake in lung and brain cancer cell lines. *Journal of Nuclear Medicine*.

[31] Li J., Beverly L., Gray B., Pak K., Ng C. (2020). Effect of fructose and glucarate on 18F-fluoroglucaric acid uptake in cancer cells. *Journal of Nuclear Medicine*.

[32] Shao D., Tian R. (2015). Glucose transporters in cardiac metabolism and hypertrophy. *Comprehensive Physiology*.

[33] Douard V., Ferraris R. P. (2008). Regulation of the fructose transporter GLUT5 in health and disease. *American Journal of Physiology. Endocrinology and Metabolism*.

[34] Savill C. M., Ayad S. R. (1983). The uptake of fibronectin or fibronectin degradation products by the nuclei of cultured cells. *Biochemical Society Transactions*.

[35] Chen C., Li R., Ross R. S., Manso A. M. (2016). Integrins and integrin-related proteins in cardiac fibrosis. *Journal of Molecular and Cellular Cardiology*.

[36] Jane-Lise S., Corda S., Chassagne C., Rappaport L. (2000). The extracellular matrix and the cytoskeleton in heart hypertrophy and failure. *Heart Failure Reviews*.

[37] Nawata J., Ohno I., Isoyama S. (1999). Differential expression of *α*1, *α*3 and *α*5 integrin subunits in acute and chronic stages of myocardial infarction in rats. *Cardiovascular Research*.

[38] Casscells W., Kimura H., Sanchez J. A., Yu Z. X., Ferrans V. J. (1990). Immunohistochemical study of fibronectin in experimental myocardial infarction. *The American Journal of Pathology*.

[39] Knowlton A. A., Connelly C. M., Romo G. M., Mamuya W., Apstein C. S., Brecher P. (1992). Rapid expression of fibronectin in the rabbit heart after myocardial infarction with and without reperfusion. *The Journal of Clinical Investigation*.

[40] Holmbom B., Naslund U., Eriksson A., Virtanen I., Thornell L. E. (1993). Comparison of triphenyltetrazolium chloride (TTC) staining versus detection of fibronectin in experimental myocardial infarction. *Histochemistry*.

[41] Sabatasso S., Mangin P., Fracasso T., Moretti M., Docquier M., Djonov V. (2016). Early markers for myocardial ischemia and sudden cardiac death. *International Journal of Legal Medicine*.

[42] Willems I. E., Arends J. W., Daemen M. J. (1996). Tenascin and fibronectin expression in healing human myocardial scars. *The Journal of Pathology*.

[43] Cheng J. D., Chen Y. C., Hu B. J. (1999). A preliminary immunohistochemical study of fibronectin in viral myocarditis. *Fa Yi Xue Za Zhi*.

[44] Hu B. J., Chen Y. C., Zhu J. Z. (2002). Study on the specificity of fibronectin for post-mortem diagnosis of early myocardial infarction. *Medicine, Science, and the Law*.

[45] Li W. S., Gong Q. J., Lu L. W., Zhang Y. J., Cheng J. D. (2006). Fibronectin immunohistochemical staining for diagnosing sudden death caused by viral myocarditis. *Fa Yi Xue Za Zhi*.

[46] Panizo A., Pardo F. J., Lozano M. D., de Alava E., Sola I., Idoate M. A. (1999). Ischemic injury in posttransplant endomyocardial biopsies: immunohistochemical study of fibronectin. *Transplantation Proceedings*.

[47] Trial J., Baughn R. E., Wygant J. N. (1999). Fibronectin fragments modulate monocyte VLA-5 expression and monocyte migration. *The Journal of Clinical Investigation*.

[48] Frangogiannis N. G., Kovacic J. C. (2020). Extracellular matrix in ischemic heart disease, part 4/4: JACC focus seminar. *Journal of the American College of Cardiology*.

