# Supplementary material for: Positron Emission Tomography (PET) with 18F-FGA for Diagnosis of Myocardial Infarction in a Coronary Artery Ligation Model
Source: Mol Imaging. 2022 Feb 9;2022:9147379. doi: 10.1155/2022/9147379 (PMC8865857; doi:10.1155/2022/9147379)
Supplement: Supplementary Materials — Supplemental information and original unedited pictures of Western blots are provided. [file 9147379.f1.docx]

**Supplemental Material**

**Positron Emission Tomography (PET) with 18F-FGA for Diagnosis of Myocardial Infarction in a Coronary Artery Ligation Model**

Vibhudutta Awasthi, Hariprasad Gali, Andria F. Hedrick, Huining Da, Venkateswararao Eeda, and Diwakar Jain,

^1^Department of Pharmaceutical Sciences, College of Pharmacy, University of Oklahoma Health Sciences Center, Oklahoma City, Oklahoma, USA

^2^Hexakit, Inc., 505 NE 46^th^ Street, Oklahoma City, Oklahoma, USA

^3^Westchester Medical Center, 100 Woods Road, Valhalla, NY 10595

**____________________________________________________________________________**

**Synthesis of Biotin-labeled glucaric acid.**

Biotin-PEG4-N-hydroxysuccinimide ester was purchased from Click Chemistry Tools (Scottsdale, AZ), D-(+)-glucosamine hydrochloride was purchased from MP Biomedicals (Irvine, CA). NMR spectra were recorded on a Varian spectrometer (Palo Alto, CA) in suitable deuterated solvents. The solvent and measurement frequency used are indicated for each experiment. The signal of the residual deuterated solvent relative to tetramethylsilane was used as the internal reference. All reaction yields are uncorrected for purity.

**Scheme S1. Synthesis of N-biotin-2-amino-glucaric acid (BGA) probe**

**Reagents & conditions:** i) CH_3_OH, NaHCO_3_ solution 24 h at room temp; ii) FGA-kit for 10 min

**Figure S1:** **Differentiation of H9c2 cells into myocytes and H_2_O_2_ treatment of mature H9c2 cells**We confirmed the maturation of H9c2 cardiomyoblasts into phenotypically mature cardiomyocytes under Pagano’s culture conditions (Pagano, M., et al, J. Cell. Physiol. 2004, 198: 408–416). H9c2 cells were grown in DMEM supplemented with 10% FBS, penicillin (100 IU), streptomycin (100 µg/ml), and sodium pyruvate (1 mM) at 37 °C in 5% CO2 environment. To induce cell differentiation, DMEM was adjusted to 1% FBS. Cell proliferation was detected by the BrdU assay (Cell Signaling Technologies). Differentiation of cells was verified by microscopy of DAPI stained cells. As shown in Fig. S1a, BrdU incorporation in H9c2 cells was reduced as the cells matured when cultured in low FBS DMEM. H9c2 cells were also grown in DMEM containing 10% FBS for 2 and 6 days. Reduced BrdU uptake in mature cells is indicative of the reduced DNA replication as the cells transitioned into their non-proliferative and mature state. Comparisons were done with one-way ANOVA and Tukey’s multiple comparison test (****P< 0.0001). The morphology of the H9c2 cells changed as they differentiated into cardiomyocytes. After one week of exposure to low FBS media, multinucleated myotubules (arrows, Fig. S1b)) were observed. To induce necrotic cell death, confluent H9c2 cells were treated with 50 µM H_2_O_2_ for 30 min (Fig. S1c).

**Figure S2: Subcellular fraction responsible for glucaric acid uptake in necrotic cells.** Biotin-labeled glucaric acid (BGA) was added to H9c2 cells undergoing H2O2-induced necrosis. BGA in (a) nuclear, (b) mitochondrial, and (c) cytosolic fractions was traced by streptavidin-HRP blotting. Purity of fractions was assessed by immunoblotting for GAPDH (37k red), COX (17k), and histone (15k) proteins in the fractions (lower panel). Actin (43k) was a common marker in all fractions. Data from three independent experiments are shown.

**Figure S3: Original digital images of the gels corresponding to Figure 6 of the main text.**

**Figure S4:** Original digital images of the gels corresponding to Figure 7 of the main text.

**Table S1:** Report for OUSHC College of Pharmacy, Oklahoma City, OK from Clarkson University Protein Core Facility. Two protein spots (2 identified) sent from Kendrick Labs, Inc.

| **Table S2:** Biodistribution of ^18^F-FGA in healthy female CD-1 mice at 15 min, 1 hr, and 2 hr p.i. Data are presented as mean + SD (n = 6). | | | | | | | | |
| --- | --- | --- | --- | --- | --- | --- | --- | --- |
| **Organ** | **15 min** | | **1 h** | | | **2 h** | | |
|  | **%ID** | **%ID/g** | | **%ID** | **%ID/g** | | **%ID** | **%ID/g** |
| **Blood** | 8.60 ± 1.60 | 5.68 ± 1.13 | | 0.25 ± 0.09 | 0.16 ± 0.05 | | 0.10 ± 0.07 | 0.06 ± 0.05 |
| **Muscle** | 11.2 ± 2.6 | 1.07 ± 0.33 | | 0.47 ± 0.17 | 0.04 ± 0.02 | | 0.66 ± 0.57 | 0.06 ± 0.05 |
| **Bone** | 6.25 ± 0.97 | 2.36 ± 0.43 | | 3.78 ± 3.50 | 1.37 ± 1.21 | | 1.82 ± 0.68 | 0.64 ± 0.22 |
| **Brain** | 0.14 ± 0.04 | 0.32 ± 0.07 | | 0.03 ± 0.04 | 0.06 ± 0.08 | | 0.03 ± 0.04 | 0.06 ± 0.09 |
| **Heart** | 0.00 ± 0.00 | 0.00 ± 0.00 | | 0.01 ± 0.00 | 0.06 ± 0.03 | | 0.00 ± 0.00 | 0.00 ± 0.00 |
| **Lung** | 0.20 ± 0.30 | 1.13 ± 1.75 | | 0.03 ± 0.01 | 0.15 ± 0.04 | | 0.02 ± 0.01 | 0.10 ± 0.02 |
| **Liver** | 2.31 ± 0.32 | 1.59 ± 0.23 | | 0.44 ± 0.12 | 0.31 ± 0.08 | | 0.35 ± 0.10 | 0.23 ± 0.07 |
| **Spleen** | 0.13 ± 0.02 | 1.31 ± 0.28 | | 0.01 ± 0.00 | 0.09 ± 0.04 | | 0.01 ± 0.01 | 0.09 ± 0.10 |
| **Kidney** | 24.4 ± 8.6 | 62.2 ± 20.4 | | 1.36 ± 0.26 | 3.70 ± 0.80 | | 0.40 ± 0.16 | 1.38 ± 1.06 |
| **Stomach** | 0.55 ± 0.05 | 0.94 ± 0.26 | | 0.10 ± 0.08 | 0.18 ± 0.20 | | 0.42 ± 0.84 | 0.72 ± 1.45 |
| **Intestine** | 0.00 ± 0.00 | 2.18 ± 0.44 | | 1.14 ± 0.42 | 0.47 ± 0.18 | | 0.00 ± 0.00 | 2.03 ± 2.73 |
